# Cases of Aneurism
*Provincial Medical Gazette, No. 1.


**Published:** 1829-10-01

**Authors:** 


### XXXVII.

### BRISTOL INFIRMARY.

### CASES OF ANEURISM.*

Mr. Morgan, apothecary to the above institution, has recorded some interesting cases of aneurism in the first, and hitherto only number of our Provincial contemporary. We cannot omit to notice them.

*Rupture of a Popliteal Aneurism—Amputation.*

*Case* 1. A healthy sailor, ætat. 30, had experienced a little pain in his right ham for some days, when he noticed there a pulsating tumour, the size of a walnut. On the fourth day from this he felt something burst in it du-

* Provincial Medical Gazette, No. I.

ring an ordinary fit of sneezing, and his leg immediately swelled and became painful. The swelling and pain increased, and in two or three days gangrene commenced in the instep and foot and quickly spread upwards, accompanied with severe constitutional irritation. Amputation was performed on the 7th day from the bursting of the aneurism, and the 11th from the day the man first became sensible of its existence; the case has since done well.

"On examining the amputated limb, an aneurismal sac was discovered, capable of containing four or five ounces of blood, originating from the middle of the popliteal artery posteriorly, about an inch of which opened into it. It was imperfectly formed behind, and presented there two openings, each readily admitting a couple of fingers, through which the contents had escaped: an abundance of blood was extravasated between the muscles and beneath the integuments in all directions. The sac elsewhere was firm and strong, lined with thick and dense laminæ of fibrine, and manifestly of much earlier date than the patient's history supposes. *The popliteal vein was filled by a firm coagulum for the space of two inches, quite obstructing it.*"

Why the latter piece of information is so carefully put in Italics, we really cannot conceive. Does Mr. Morgan imagine that it bears upon any disputed point? or does he actually think it wonderful that the circulation should be obstructed in the popliteal vein, when a large solid sac originated from the posterior part of the popliteal artery, and "abundance of blood" was extravasated in every direction? It seems to us a very natural occurrence.

*Case* 2. A cattle-drover, ætat. 32, was admitted into the infirmary under Mr. Richard Smith, with the left lower extremity twice the size of the other, as high as the lower third of the thigh—the foot and surface of the leg œdematous, with distinct fluctuation, as of matter beneath—considerable pain, but not much tenderness, in different parts of the leg—general health greatly impaired—restless nights, hectic, and other symptoms of constitutional irritation. He had been for many years in the habit of walking great distances, and had travelled upwards of a hundred miles in two days, very shortly before he was taken ill. The disease commenced two months before his admission with considerable pain in the calf of the leg, which shortly confined him to bed, and was followed by œdematous swelling about the ankle. The pain and the swelling increased, and his health became disturbed with their advance.

"This condition of things had been gradually coming on from the first: he was not, like the former man, sensible of any sudden bursting, neither at any time was he conscious of the existence of a distinct tumour, or pulsation. It was determined, in consultation, first, to puncture the most fluctuating part, and to be guided by the result in forming an opinion on the practice to be pursued: an opening accordingly was made about the middle of the calf, but nothing escaped. By introducing the finger, an abundance of soft coagulum could be felt, occupying a large cavity within, thus clearing up a little of the obscurity in which the disease previously was involved. Amputation was now immediately performed, and the following state of parts discovered on examining the limb: an aneurismal sac, large enough to hold twelve or fourteen ounces of blood, arose from the posterior part of the middle of the popliteal artery, about two inches of which communicated with it. Anteriorly and laterally it was distinct and strong, but very defective posteriorly, where numerous breaches existed through which its contents could find a ready issue. The sac, such as it was, contained a quantity of old coagulum, but not much laminated fibrine—the popliteal vein was pervious throughout. An immense quantity of extravasated blood, much more than in the former case, was found in various parts of the leg, particularly between the muscles; and here and there a little uncircumscribed purulent effusion existed, the result, probably, of diffused cellular inflammation.

"The patient is now in the house, with a favorable prospect of recovery."

CASE 3. *Spontaneous Cure of Femoral Aneurism—Gangrene.*

Morris Ryland, æt. 35, plethoric, and enjoying good health, was admitted Jan. 2d, 1817, under the care of Mr. Lowe, with an aneurismal tumour of the right femoral artery, half way down the thigh. It was the size of a large orange, flattened, not well defined, situated deeply, and painful—pulsation considerable—limb hot—no material interruption to its functions—pulse accelerated—much excitement. He had felt a pulsation for more than two months, but only noticed the tumour a week. Perfect rest, low diet, V. S. to twenty-four ounces, free purging, and an evaporating lotion to the thigh, were had recourse to, and in forty-eight hours after his admission, all pulsation had evidently ceased. The foot, however, was cold and numb, and the toes evinced a disposition to gangrene, though no swelling of the thigh, nor extraordinary diminution of the tumour, were present to indicate bursting of the tumour.

"The treatment now consisted of the application of heat to the limb, and repeated gentle friction. The progress of the case was marked chiefly by very slow sphacelus of part of the foot. At the expiration of four months, the toes and soft parts of the metatarsus had sloughed off, leaving the metatarsal bones dead and protruding, surrounded by a languid and ill-conditioned wound. The man's health had materially suffered by this protracted confinement; he experienced considerable pain in the foot; from long continuance in the same posture, his knee had become permanently bent; and the prospect of recovery was remote and uncertain: all these circumstances rendered amputation not only desirable but necessary, and the leg accordingly was removed below the knee, on April 29th. The three arteries bled freely, and were rather enlarged. The stump healed very slowly, and by granulation alone. The patient's general health gradually improved, and he was at length discharged quite well, undertaking the situation of a book-keeper at one of our principal coach offices, which he still holds with uninterrupted good health. The aneurismal tumour when he left the Infirmary, Sept. 2, was diminished two-thirds of its former size, and never was found to pulsate again after its first ceasing."

The above case is not strictly an instance of the *spontaneous* cure of aneurism, for the cessation of pulsation in the tumour, &c. followed starvation, a bleeding to twenty-four ounces, free purging, and the other items of pretty "heroic" antiphlogistic treatment. One might just as well talk of the *spontaneous* cure of a pneumonia, after salivation with calomel and opium and the active use of the lancet. We candidly avow that we are not satisfied with the treatment pursued in this case. We do not see the necessity for starting with such sudden and violent treatment as was practised, and no earthly doubt can exist that the four months' suffering from tedious gangrene, the consequent cachexia, and the ultimate amputation, were its unfortunate consequences. God forbid that we should give utterance to any reflections injurious to the character, or grating to the feelings of a brother practitioner. We know the uncertainties of our science and the difficulties besetting its professors, but where we conceive that speaking out would be of benefit, it becomes an unpleasant duty to do so. Aneurism, it should be recollected, depends, in the majority of cases, on a morbid state of the arterial system, and not unfrequently of the heart also. It is obvious that arteries thus altered are weakened, and that gangrene is infinitely more likely to take place in patients so affected, than in those whose circulating system is sound. We put it then to any reflecting man, whether large abstractions of blood, and sudden or violent depleting measures, are not to be avoided under these circumstances by all who wish the welfare of their patients, or are chary of their own reputation. Frequent small bleedings are of service when the loss of blood is required, but large ones are often injurious, always unsafe.

CASE 4. *External Iliac Aneurisms—Bronchitis—Other Diseases—Operation—Death.*

The following case must give rise to some observations.

A man, æt. 63, was admitted under Mr. Daniel, with a large tumour behind and below the right Poupart's ligament, projecting much beyond the level of the adjoining parts, pulsating strongly, very painful, and impeding the motion of the leg, although he had just walked a mile without assistance. The limb was very œdematous—the foot rather numb—the action of the heart irregular, and exceeding the natural standard both in frequency and power—some chronic bronchitis, to which he was subject—health otherwise tolerably good. The disease had commenced seven or eight months previously, but had produced little inconvenience, till within the last three weeks, when its increase had been more rapid, and the pain and œdema had appeared. He was bled to 16 ounces on the day of his admission, and the operation proposed on the next, but he would not consent till the 7th November, (date of admission not stated,) when the symptoms had much increased in severity. The pulse was powerful and averaged 100, the pain very great. During the interval in question he had again been bled to 16 ounces, kept quiet, regulated in his bowels, and treated with an anodyne every night. On the 7th, the Abernethian operation was performed, apparently with dexterity and ease. The remainder we shall give in ipsissimis verbis of the reporter.

"The result of the operation, so far as respected the aneurismal disease, was very gratifying; the pain soon permanently ceased; the œdema in three or four days had nearly subsided; the temperature and sensibility of the limb were very little affected, and not the least disposition to gangrene was observed. Unfortunately, however, a severe attack of bronchitis succeeded, to which, notwithstanding every attention, he fell a victim on the eleventh day. He had likewise some inflammation of the abdominal viscera in the neighbourhood of the wound. The pulse was always full and strong, and more irregular than at first: the pulsation of the abdominal aorta and common iliacs was very forcible. He never complained of head-ache, but was much dejected, and prone to gloomy forebodings. The inflammatory affections were combated by the abstraction of seventy-two ounces of blood at five different times, (which was always much inflamed,) and the application of numerous leeches to the abdomen, and two large blisters to the chest, aided by appropriate medicines and a restricted diet. He had become much better, and was considered by no means in immediate danger, when, on the morning of the eleventh day, in a gentle fit of coughing, he expired. The tumour had not perceptibly diminished, and felt firm and solid throughout.

"The following appearances were observed in the examination of the body. Traces of rather considerable inflammation existed at the lower part of the abdomen internally, especially on the right side. The ascending colon was strongly adherent to the peritoneum anteriorly, and its coats were diseased. The right psoas muscle was the seat of an abscess of some magnitude, unconnected with any other part. The ligature had been applied one inch and a half from the commencement of the external iliac artery, and about the same distance from the aneurism: it was on the point of being detached. The artery above it was closed, and filled by a firm clot as high as its origin, and every thing about it was in a healthy state down to the seat of the disease. The aneurismal tumour was the size of a cocoa-nut, deeply imbedded in the hollow of the groin, and in immediate contact with the head of the femur, which was slightly carious. Poupart's ligament was tightly stretched across it in front. The upper and posterior part of the sac had given way, and a large quantity of blood was extravasated behind the fascia iliaca. The arterial system in general was much diseased. The opposite external iliac was crowded with calcareous deposits, and the femoral artery of the aneurismal limb had numerous opake patches, the commencement of a similar state. The ascending aorta was considerably dilated, and its parietes so soft that a slight force ruptured them. A small aneurism about the size of a walnut arose from the right subclavian artery at its origin, of which no suspicion existed during life. An arteria innominata was found on each side, the left dividing as the right into carotid and subclavian. *The lungs were universally adherent to the costal pleura, much denser than natural, and the air tubes filled with mucus.*"

How frequently is one reminded in the details of medical cases, of the Frenchman and his horse! The result of the operation was very gratifying—all was doing beautifully—not the least disposition to gangrene was evinced, and the cure would have been superb, *but*, "unfortunately," the patient—— died. He died, we are told, of acute bronchitis, but not a trace of it can we perceive on dissection, unless "the air tubes filled with mucus" be deemed the anatomical character of that disease. Perhaps an anecdote may help us out of a portion of the difficulty. Not a hundred years ago, nor a hundred miles from this metropolis, a physician was summoned to a patient, whom her medical attendant (an M. D.) pronounced to be labouring under INFLAMMATORY ASTHMA! This new disease was found to consist, on examination, in orthopnœa, dyspnœa, cough, and the other symptoms of *organic disease of the heart*, which was manifestly present. Is it *possible* that the present patient had a touch of the *inflammatory asthma*,

"I thank thee, Jew, for teaching me that word."?

Certainly he had the symptoms of hypertrophy, and probably dilatation of the heart, accompanied with Winter cough, one of its most frequent concomitants in old persons, though not a syllable is said respecting the state of the organ on dissection. The universal adhesions of the pleuræ, old ones we suppose, and consolidated state of lungs, are met with in nine out of ten examinations of patients, dying of diseases of the heart. The "rather considerable" peritoneal inflammation in the neighbourhood of the wound, and abscess in the contiguous psoas, were no mean items in the case, and helped to shuffle off the mortal coil, no doubt. So much for the post-mortem examination, but a word or two remains to be said upon circumstances that occurred before the curtain dropt before the scene.

A patient of 63, with chronic bronchitis and suffering from symptoms not unlike those of diseased heart, appears to us to have been no proper subject for the operation of tying the external iliac artery. Do they keep no stethoscope in the Bristol Infirmary, for auscultation appears to have never been dreamt of? If they will not use the ears with which Providence has endowed them we would earnestly advise the medical attendants to purchase the instrument as soon as may be, and the last edition of Laennec. This patient lost thirty-two ounces of blood from the arm before the operation, and seventy-two after it, making in all one hundred and four ounces from a man of sixty three, whose age, independent of the aneurism, would almost ensure a diseased arterial system; who suffered habitually from bronchitis; and laboured under irregularity in the action of the heart. Is it wonderful that he should "expire in a gentle fit of coughing"?

In order to shew that provincial surgeons "are not tardy in following their metropolitan brethren in any *real improvement*," Mr. Morgan mentions a case of "supposed aneurism of the arteria innominata," which "seemed a very fair one for tying the subclavian and carotid arteries, according to the principle lately revived by Mr. Wardrop." The patient, however, took a more judicious view of the *real improvement*, than Mr. Morgan, for he refused to submit to its performance, and has since very wisely walked off. Before concluding, we may hint that there are some other *real improvements* which might perhaps be taken under Mr. M.'s patronage; we mean, auscultation in patients aged sixty-three, affected with aneurism and symptoms of thoracic disease.

---

